# Inulanolide A as a new dual inhibitor of NFAT1-MDM2 pathway for breast cancer therapy

**DOI:** 10.18632/oncotarget.8873

**Published:** 2016-04-20

**Authors:** Jiang-Jiang Qin, Wei Wang, Sushanta Sarkar, Sukesh Voruganti, Rajesh Agarwal, Ruiwen Zhang

**Affiliations:** ^1^ Department of Pharmaceutical Sciences, School of Pharmacy, Texas Tech University Health Sciences Center, Amarillo, TX 79106, USA; ^2^ Cancer Biology Center, School of Pharmacy, Texas Tech University Health Sciences Center, Amarillo, TX 79106, USA; ^3^ Department of Pharmaceutical Sciences, Skaggs School of Pharmacy and Pharmaceutical Sciences, University of Colorado Denver, Aurora, CO 80045, USA; ^4^ University of Colorado Cancer Center, University of Colorado Denver, Aurora, CO 80045, USA

**Keywords:** Inulanolide A, NFAT1-MDM2 pathway, p53-independent, breast cancer orthotopic tumor model, metastasis

## Abstract

The transcription factor NFAT1 and the oncogene *MDM2* have crucial roles in breast cancer development, progression, and metastasis. We have recently discovered that NFAT1 activates MDM2 expression. Here, we identified a small molecule (named Inulanolide A) that dually inhibited both NFAT1 and MDM2 in breast cancer cells *in vitro* and *in vivo*. Unlike conventional MDM2 inhibitors, Inulanolide A (InuA) exerted its selective anticancer activity in both p53-dependent and -independent manners. InuA decreased cell proliferation and induced G2/M phase arrest and apoptosis in breast cancer cells; it also led to a decrease in MDM2, NFAT1 and proteins associated with cell proliferation, and an increase in apoptotic signal related proteins. In a mouse orthotopic model, JapA suppressed tumor growth and lung metastasis without host toxicity. Thus, InuA is a novel NFAT1 and MDM2 dual targeting agent and may be a clinical candidate for breast cancer therapy. This study also validates the effectiveness of dually targeting NFAT1 and MDM2 in breast cancer.

## INTRODUCTION

Breast cancer remains a major global health challenge [1–3]. The development of targeted therapies, including estrogen receptor (ER) modulators and human epidermal growth factor receptor 2 (HER2) antagonists, has improved the mortality of this disease [4, 5]. Despite these recent clinical advances, intrinsic and acquired resistance to these therapies is still common. Some subtypes of breast cancer, such as triple negative breast cancer (TNBC), cannot benefit from the existing targeted therapies due to the intrinsic lack of molecular targets and their highly aggressive nature [6–9]. Therefore, developing effective and safe therapy for advanced breast cancer is a highly unmet medical need.

Mouse Double Minute 2 (MDM2) promotes cancer cell proliferation and cell cycle progression, inhibits DNA damage response, reduces apoptosis, and stimulates metastasis [10–12]. The *MDM2* oncogene is genetically amplified and/or overexpressed in breast cancer patients [13–15] and is positively associated with poor prognosis and high incidence of metastasis [16–20]. Therefore, this multifaceted oncogene has been suggested as a promising molecular target for breast cancer therapy. Several targeting strategies have been developed to inhibit MDM2 and its activity, leading to the discovery of a number of MDM2 inhibitors [21–28]. We have recently demonstrated that the transcription factor NFAT1 transactivates MDM2, independent of p53 [29]. NFAT1 is aberrantly activated and overexpressed in breast cancer cells and promotes breast cancer development and progression [30–32]. Therefore, dually targeting MDM2 and NFAT1 could be a novel and effective approach to breast cancer therapy.

We have recently proposed to develop dual inhibitors of NFAT1-MDM2 pathway and discovered a novel class of naturally occurring dimeric sesquiterpenoids, including the lead compounds JapA [26, 33] and Inulanolide A (InuA). JapA has been demonstrated as a potent and specific dual NFAT1-MDM2 inhibitor and has shown excellent anticancer activity *in vitro* and *in vivo* [26, 33]. As for every new drug investigation, there is no guarantee that one compound will be the clinical lead for future clinical studies. Therefore, we have selected several backup compounds. In addition, to further determine the efficacy and safety profiles of this class of natural NFAT1-MDM2 dual inhibitors and to explore the underlying mechanisms of action and structure-activity relationship (SAR), it is necessary to evaluate InuA and other candidates, which have different chemical structures but show similar activities. The present study was designed to investigate the anticancer efficacy of InuA and its molecular mechanisms of action *in vitro* and *in vivo*. In the initial screening for *in vitro* cytotoxicity of InuA, breast cancer cells exhibited high sensitivity to this compound. Therefore, we utilized breast cancer models for further evaluation of this compound. Our results demonstrate the therapeutic potential of targeting NFAT1-MDM2 pathway and provide new insights into MDM2 targeting strategies, suggesting that InuA may be a novel therapeutic agent for the treatment and prevention of human breast cancer.

## RESULTS

### InuA exhibits selective cytotoxicity toward different types of cancer cells, with minimal effects on normal cell growth

**Figure 1:**
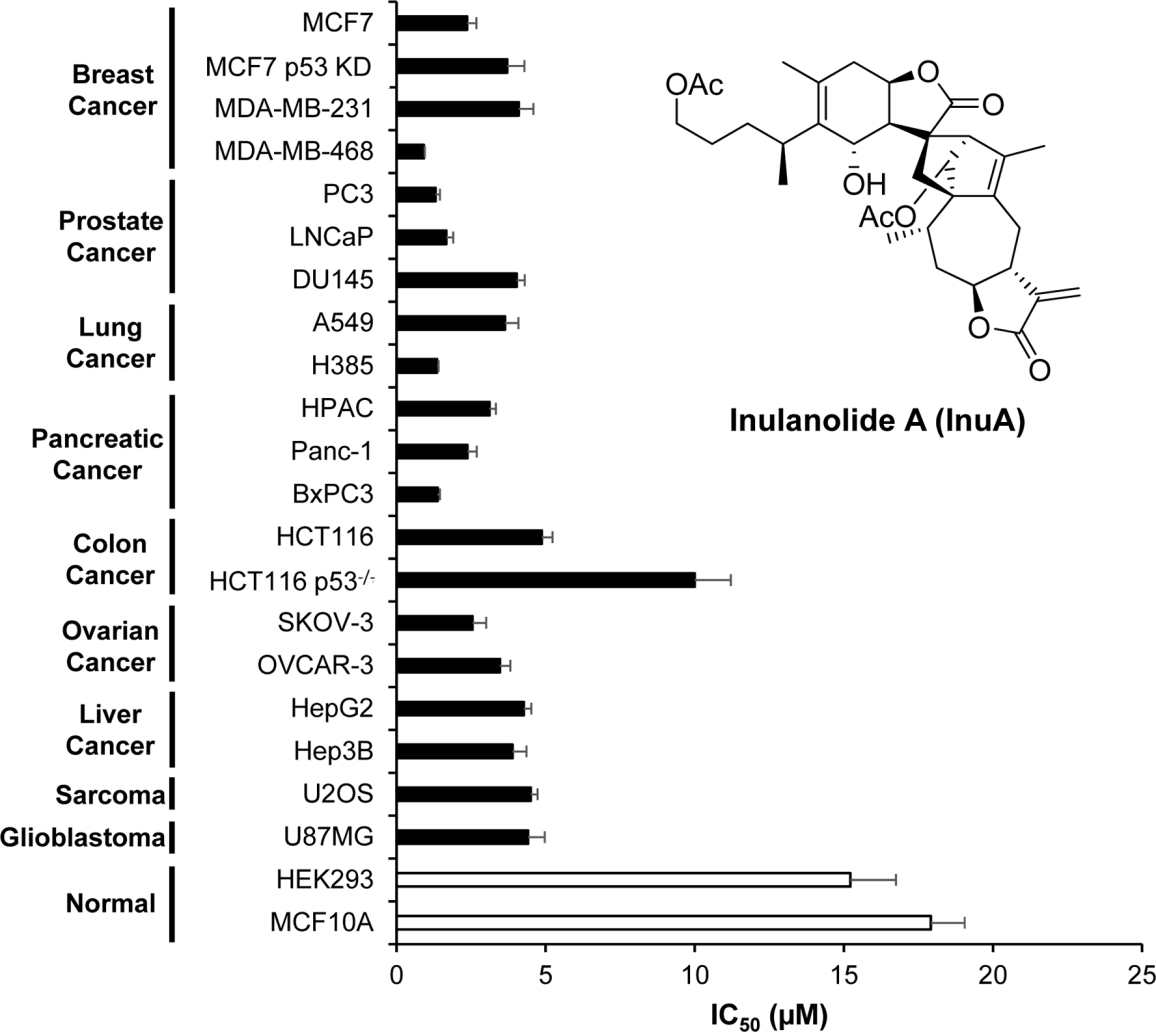
Cytotoxicity of InuA against various normal and cancer cell lines Various normal and cancer cell lines were treated with InuA (0–50 μM) for 72 h. The cell viability and IC_50_ values were then determined using MTT assays. All assays were performed in triplicate and repeated three times. MCF7 p53 KD, MCF7 p53 knockdown; HCT116 p53^−/−^, HCT116 p53 knockout.

InuA was first tested for its effects on cell growth in two normal cell lines and 20 cancer cell lines representing nine types of human cancer (breast, prostate, lung, pancreatic, colon, ovarian, and liver cancer, sarcoma, and glioblastoma). After exposure of cells to various concentrations of InuA (0 to 50 μM) for 72 h, the cell viability and IC_50_ values were determined using the MTT method. InuA exhibited a broad cytotoxicity spectrum (IC_50_ values from 0.9 to 10.0 μM) against human cancer cells. Among this, breast cancer MCF7 (p53 wild-type), MCF7 p53 knockdown (KD), MDA-MB-231 (p53 mutant), and MDA-MB-468 (p53 mutant) cells exhibited strong sensitivity to InuA treatment, with the IC_50_ values of 2.4, 3.7, 4.1, and 0.9 μM, respectively (Figure 1). Most importantly, in comparison to cancer cells, the normal HEK293 and MCF10A cells were much less sensitive to InuA, suggesting that this compound has selective cytotoxicity against cancer cells (Figure 1). Interestingly, HCT116 p53^−/−^ cells (IC_50_ = 10.0 μM) and MCF7 p53 KD cells (IC_50_ = 3.7 μM) had higher IC_50_ values than their parent cells (4.9 and 2.4 μM, respectively), indicating that the anticancer effects of InuA might not be totally p53-indepenent.

### InuA exerts *in vitro* anti-breast cancer activity

**Figure 2:**
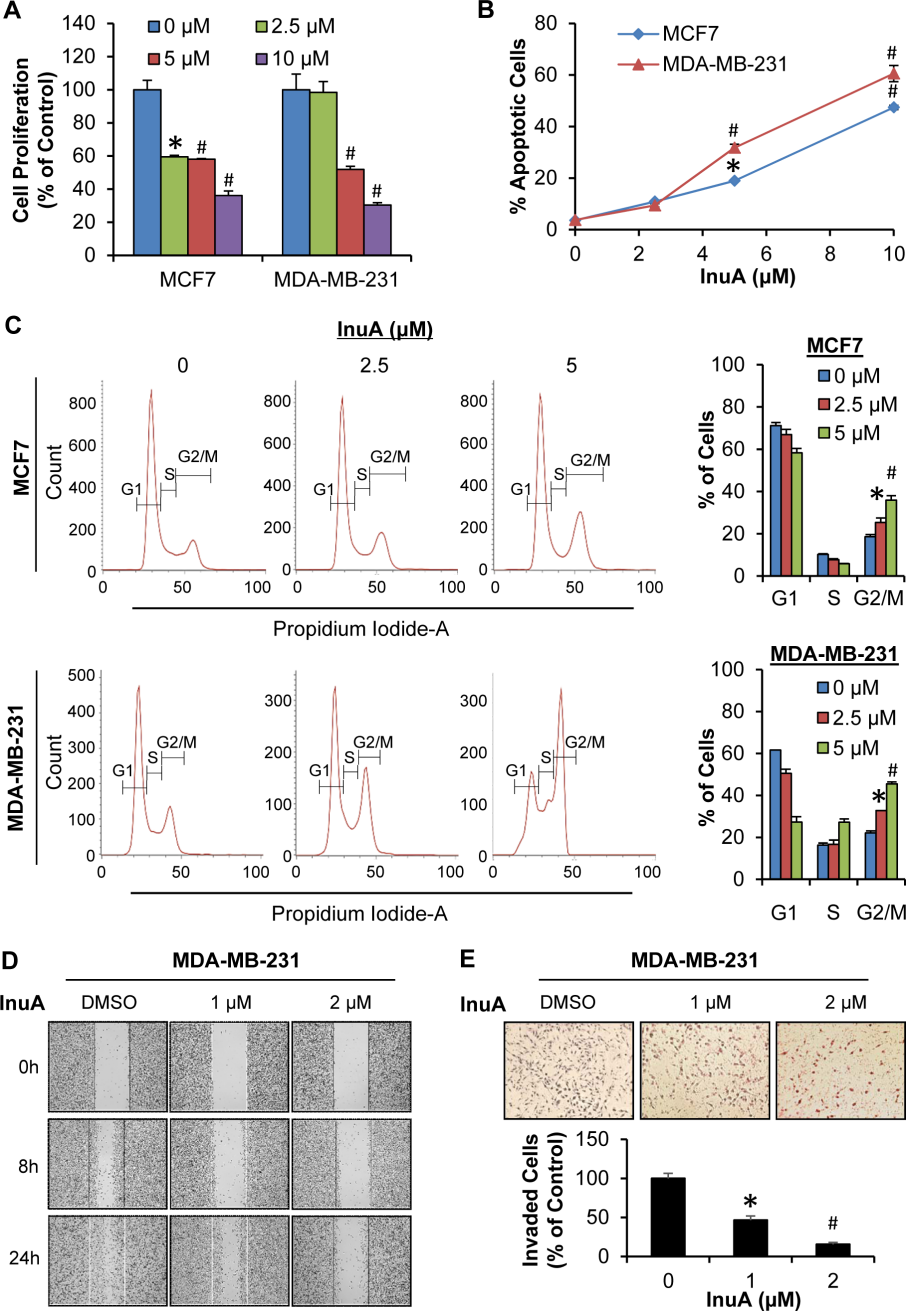
*In vitro* anti-breast cancer activity of InuA (**A**) Antiproliferative effects of InuA. MCF7 and MDA-MB-231 cells were exposed to various concentrations (0, 2.5, 5, and 10 μM) of InuA for 24 h, followed by measurement of cell proliferation via the BrdUrd assay. The proliferative index is in comparison to untreated cells; (**B**) Induction of apoptosis by InuA. MCF7 and MDA-MB-231 cells were treated with InuA (0, 2.5, 5, and 10 μM) for 48 h, followed by measurement of apoptosis using Annexin V assay/flow cytometry; (**C**) Effects of InuA on cell cycle progression. MCF7 and MDA-MB-231 cells were treated with InuA (0, 2.5 and 5 μM) for 24 h, followed by determination of cell cycle distribution using flow cytometry; (D&E) Effects of InuA on metastasis. MDA-MB-231 cells were treated with InuA (0, 1, and 2 μM) for 24 h, the migration ability was measured by a wound healing assay (**D**); and cell invasion ability was measured using a Transwell cell invasion assay (**E**). All assays were performed in triplicate and repeated three times. (*P < 0.05 and ^#^*P* < 0.01) DMSO, dimethyl sulfoxide.

As shown in Figure 2A, InuA inhibited the proliferation of both MCF7 and MDA-MB-231 cells in a concentration-dependent manner, regardless of the p53 status. Similarly, InuA induced apoptosis in both breast cancer cell lines in concentration-dependent and p53-independent manners (Figure 2B). InuA treatment also caused cell cycle arrest at G2/M phase in both cell lines (Figure 2C), with the initial effective concentration at 2.5 μM. We further examined the effects of InuA on breast cancer cell migration and invasion. As shown in Figure 2D, the control MDA-MB-231 cells migrated into almost all of the wound area by 24 h, whereas InuA significantly inhibited the cell migration in a concentration-dependent manner. Similarly, InuA at the sublethal concentrations significantly prevented the invasion of MDA-MB-231 cells (Figure 2E).

### InuA suppresses tumor growth and lung metastasis in nude mice bearing MDA-MB-231 orthotopic tumors

**Figure 3:**
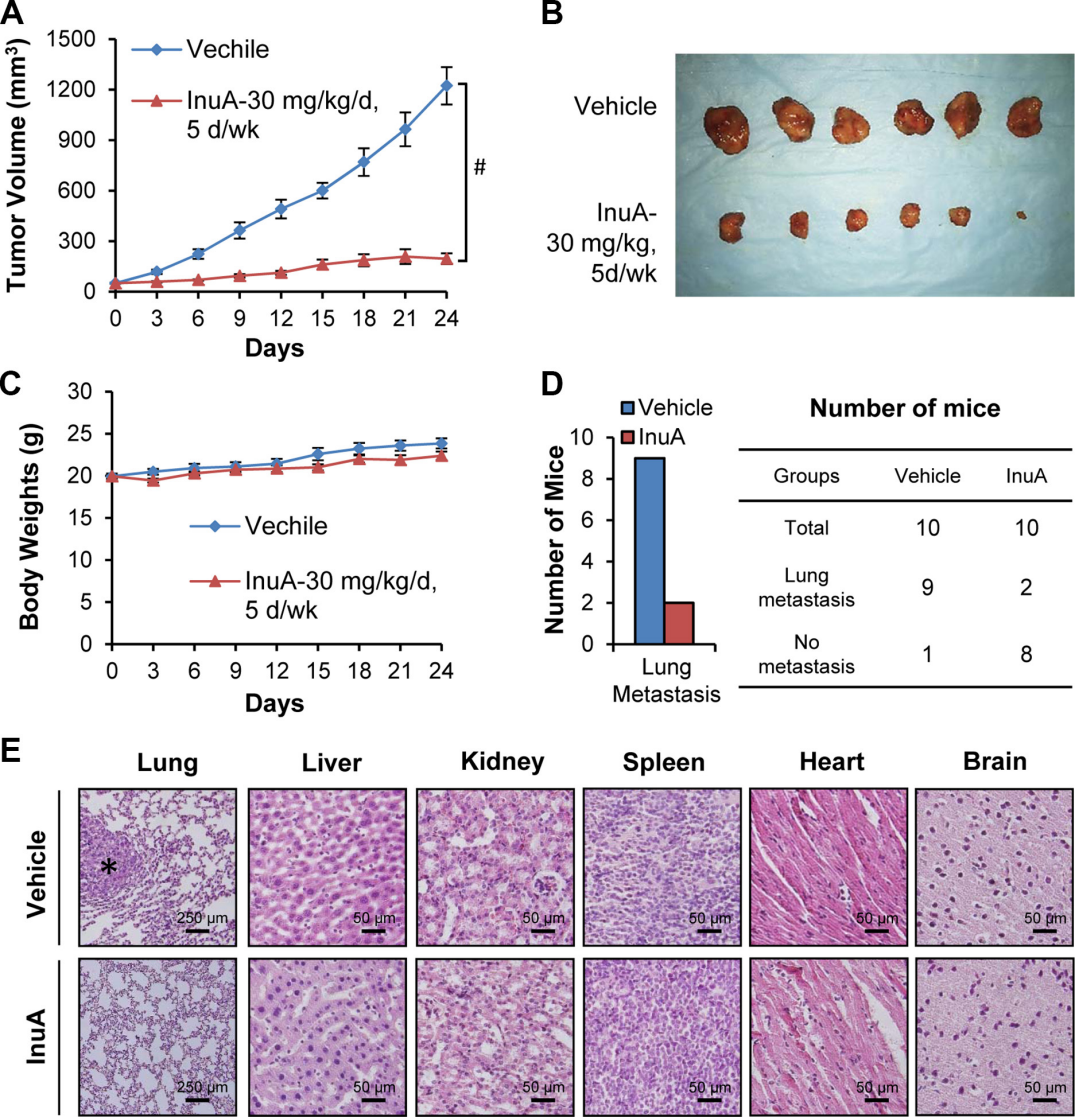
*In vivo* efficacy of InuA in nude mice bearing MDA-MB-231 orthotopic tumors (**A**) MDA-MB-231 cells were implanted orthotopically into the second thoracic mammary fat pad of nude mice. After two weeks, tumor-bearing mice were grouped and treated with InuA by intraperitoneal injection at dose of 30 mg/kg/d, 5 d/wk for 24 days. The control mice received vehicle only. (**B**) At the end of the experiments, representative tumors were removed and photographed. (**C**) The mice were monitored for changes in body weights as a surrogate marker for toxicity. (**D**) The mice were examined for tumor metastasis to various organs and the numbers of mice with metastasis to lungs were counted. (**E**) Various tissues (lung, liver, kidney, spleen, heart, and brain) were carefully removed from mice, and H & E staining of the paraffin sections of these tissues was performed (all images represent serial sections; scale bar, 50 or 250 μm). The black asterisk indicates areas of breast cancer cell invasion. (^#^*P* < 0.01).

We further investigated the *in vivo* efficacy of InuA in the MDA-MB-231 orthotopic model of human breast cancer. The compound was given by intraperitoneal injection at 30 mg/kg/d, 5 d/wk for 24 days. As shown in Figure 3A and 3B, InuA treatment significantly inhibited the tumor growth by 84% (*P* < 0.01). However, the compound did not affect the average body weights of the mice in comparison to that of control mice, indicating that there was no obvious host toxicity during the treatment period (Figure 3C). At the termination of the experiments, all mice were autopsied for assessment of the metastatic lesions in various organs. The results showed that 9 out of 10 control mice developed metastatic lesions in the lungs, while InuA treatment decreased the incidence of lung metastasis to 2/10 (Figure 3D). Lungs and other major organs (liver, kidneys, spleen, heart, and brain) were dissected for histological examinations, confirming the inhibition of lung metastasis (Figure 3E). In addition, no abnormalities were observed in other organs from these mice, suggesting the absence of any host toxicity.

### InuA inhibits NFAT1-MDM2 pathway *in vitro* and *in vivo*

**Figure 4:**
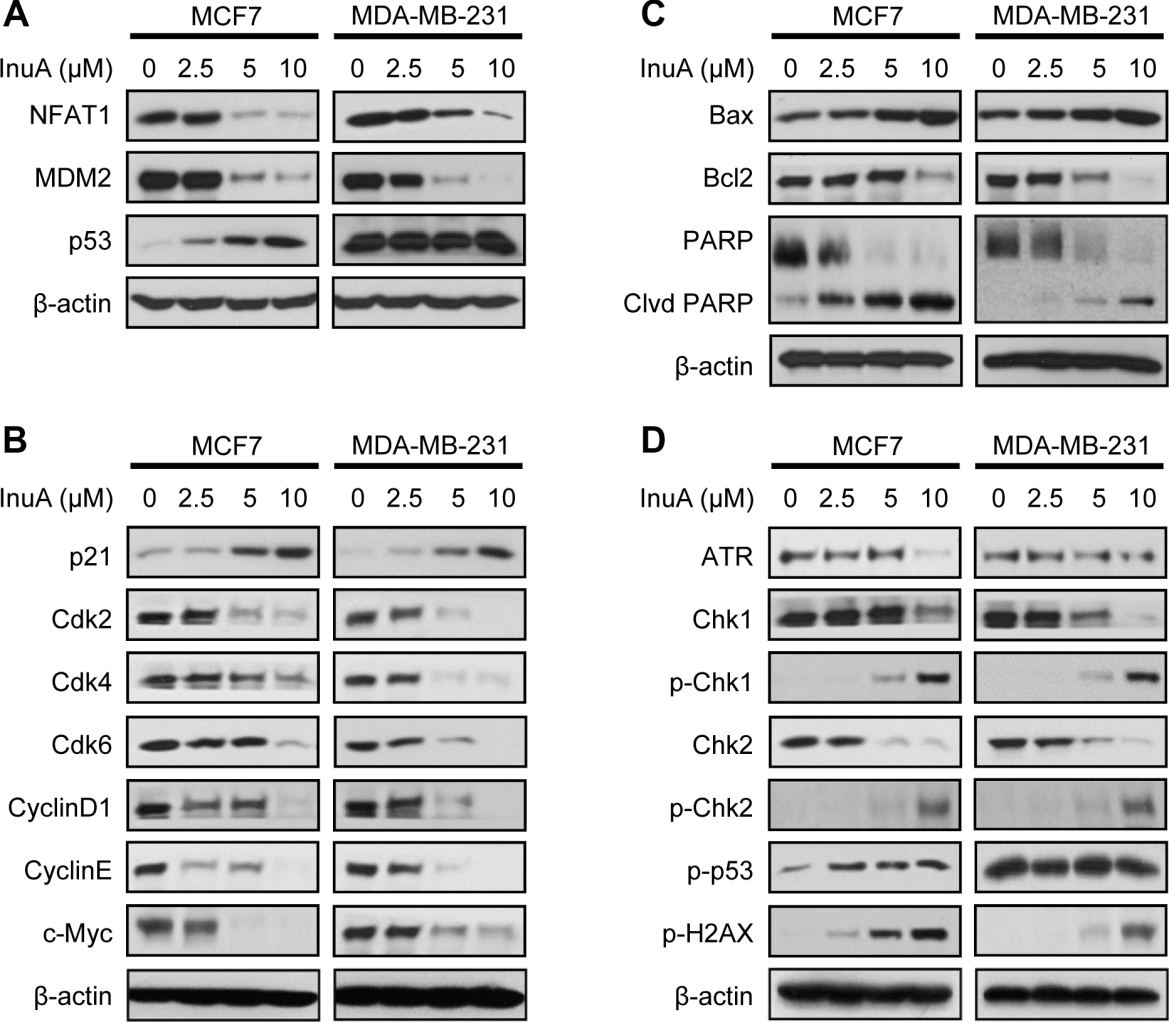
*In vitro* effects of InuA on NFAT1-MDM2 pathway and on the expression of proteins related to cell cycle progression, apoptosis, and DNA damage response MCF7 and MDA-MB-231 breast cancer cells were exposed to various concentrations of InuA for 24 h, and the expression of proteins related to (**A**) NFAT1-MDM2 pathway, (**B**) cell cycle progression, (**C**) apoptosis, and (**D**) DNA damage response were examined by Western blotting assay. All assays were performed in triplicate and repeated three times. NFAT1, nuclear factor of activated T cells 1; MDM2, mouse double minute 2.

**Figure 5:**
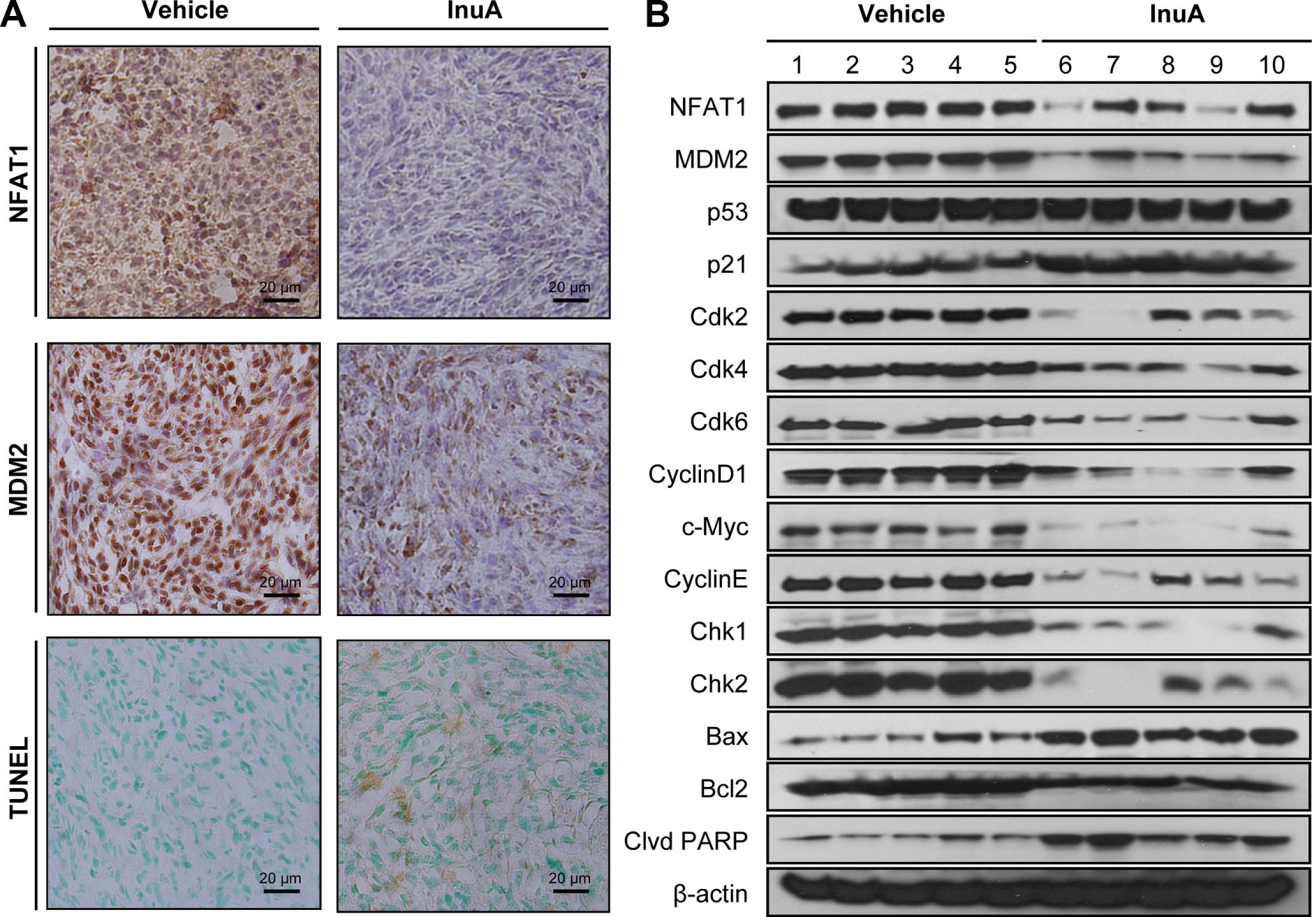
*In vivo* effects of InuA on NFAT1-MDM2 pathway and on the expression of proteins related to cell cycle progression, apoptosis, and DNA damage response MDA-MB-231 cells were implanted orthotopically into the second thoracic mammary fat pad of nude mice. After two weeks, tumor-bearing mice were grouped and treated with InuA by intraperitoneal injection at dose of 30 mg/kg/d, 5 d/wk for 24 days. The control mice received vehicle only. At the end of the experiments, the orthotopic tumors were carefully removed and analyzed by (**A**) immunohistochemistry and TUNEL staining (all images represent serial sections; scale bar, 20 μm) and (**B**) Western blotting assay (Each lane represents an individual mouse tumor sample; control mice: lanes 1–5; InuA-treated mice: lanes 6–10).

The effects of InuA on NFAT1-MDM2 pathway were first investigated in MCF7 and MDA-MB-231 breast cancer cells. As shown in Figure 4A, InuA downregulated the expression of NFAT1 and MDM2 in both cell lines and activated the expression of wild-type p53 in MCF7 cells. However, no significant changes were observed in mutant p53 in MDA-MB-231 cells. To further investigate the molecular mechanisms responsible for the anticancer activity of InuA, we evaluated its effects on the expression of proteins involved in cell cycle progression, apoptosis, and DNA damage response. As shown in Figure 4B and 4C, InuA upregulated the expression of p21 and Bax, induced the cleavage of PARP, and reduced the expression of Cdk2, Cdk4, Cdk6, Cyclin D1, Cyclin E, c-Myc, and Bcl2 in both breast cancer cell lines. The compound also inhibited the expression of ATR, resulted in the phosphorylation of Chk1 at Ser317, Chk2 at Thr68, wild-type p53 at Ser15, and H2AX at Ser139 in breast cancer cells (Figure 4D). Next, we examined the protein expression in MDA-MB-231 orthotopic tumors by immunohistochemical staining, TUNEL assay (Figure 5A), and Western blotting assay (Figure 5B). The similar results were obtained in InuA-treated tumors as those observed *in vitro*.

### Knockdown of NFAT1 and MDM2 reduces the anticancer activity of InuA

**Figure 6:**
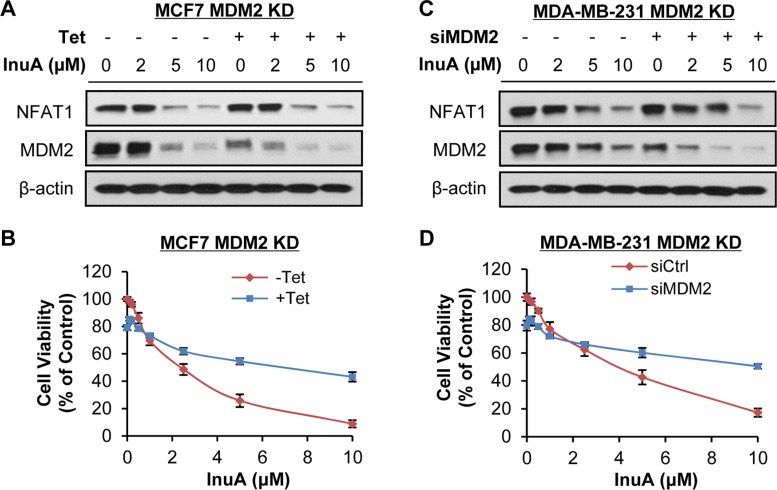
Effects of MDM2 knockdown on InuA-induced cell death The inducible MDM2 knockdown (KD) MCF7 cells were incubated with (Tet+) or without tetracycline (Tet-) for 24 h, followed by exposure to various concentrations of InuA for (**A**) 24 h for examination of expression levels of NFAT1 and MDM2; and (**B**) 72 h for cell viability determination. MDA-MB-231 cells were transfected with MDM2 siRNA or the respective control siRNA for 36 h and then treated with various concentrations of InuA for (**C**) 24 h for NFAT1 and MDM2 protein levels; and (**D**) 72 h for cell viability. All assays were performed in triplicate.

**Figure 7:**
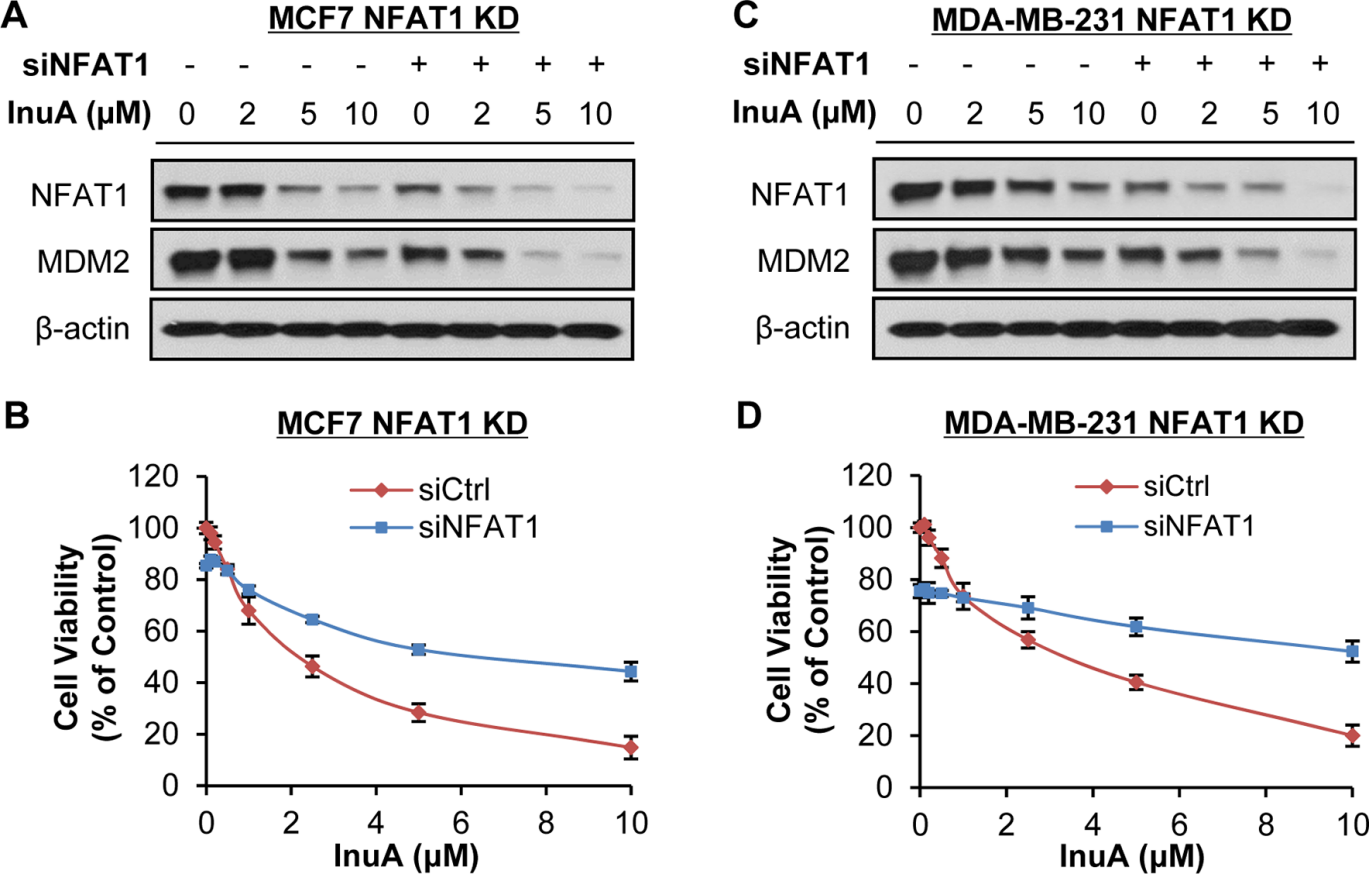
Effects of NFAT1 knockdown on InuA-induced cell death MCF7 (**A, B**) and MDA-MB-231 (**C, D**) breast cancer cells were transfected with NFAT1 siRNA or the respective control siRNA for 36 h and then treated with various concentrations of InuA for (A, C) 24 h for examination of expression levels of NFAT1 and MDM2; and (B, D) 72 h for cell viability determination. All assays were performed in triplicate.

To demonstrate the importance of NFAT1-MDM2 pathway in InuA's anticancer activity, we compared the effects of InuA on inducible MDM2 KD MCF7 cells with their parent cells. As shown in Figure 6A and 6B, tetracycline (Tet)-induced MDM2 KD inhibited cell growth and reduced the inhibitory effect of InuA on cell viability. In MDA-MB-231 cells, the transient transfection of MDM2 siRNA resulted in MDM2 KD, which decreased the cytotoxicity of InuA against cancer cells (Figure 6C and 6D). We also compared the cytotoxicity of InuA against parent and NFAT1 KD cells. As shown in Figure 7A–7D, the transient transfection of NFAT1 siRNA caused NFAT1 KD in both MCF7 and MDA-MB-231 cell lines, which further decreased the NFAT1-activated MDM2 expression and reduced the effects of InuA on cell viability. Taken together, these results indicated a critical role of NFAT1-MDM2 pathway in the InuA-induced cell death in breast cancer cells.

## DISCUSSION

**Figure 8:**
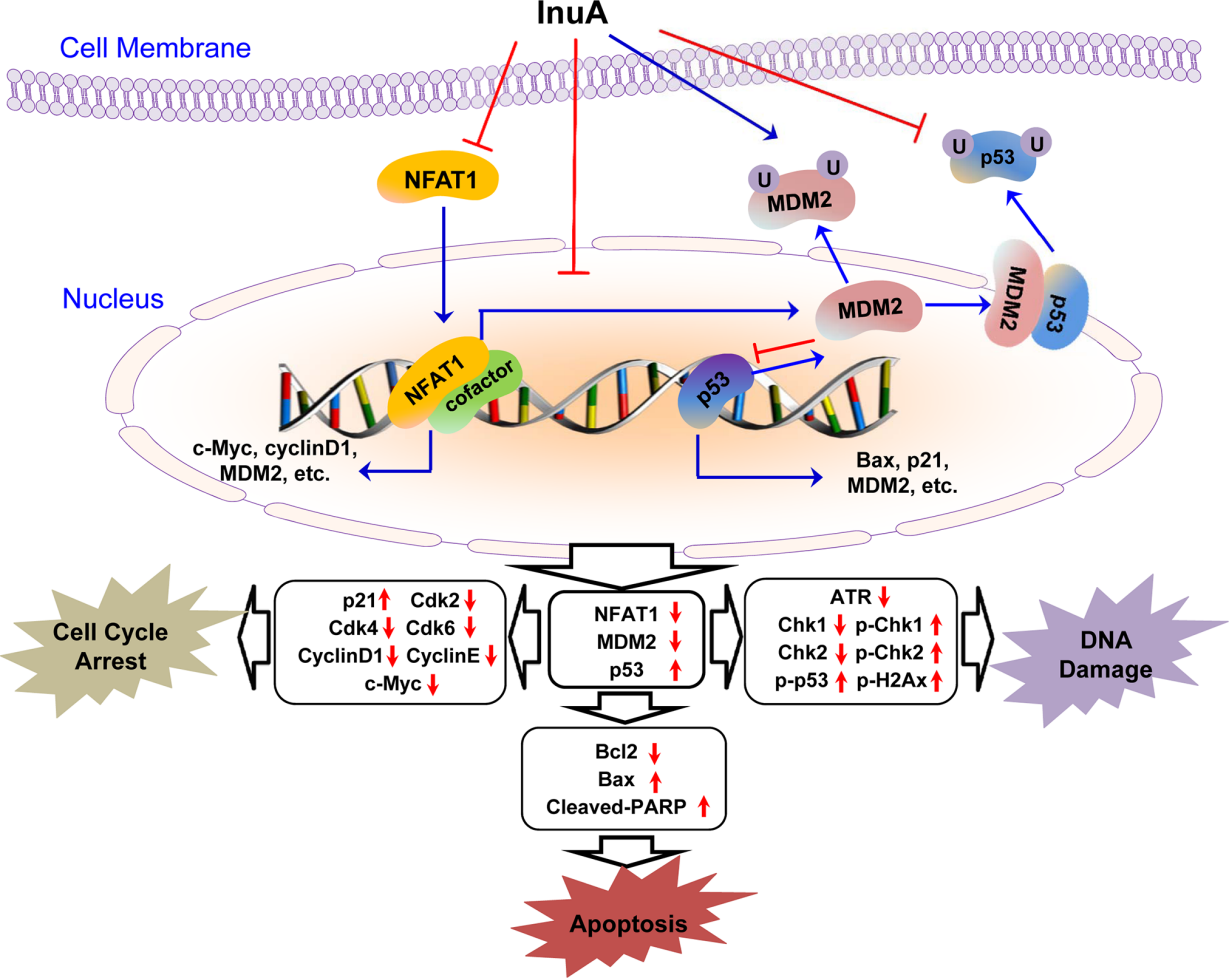
The diagram depicts the possible mechanisms of action of InuA against cancer cells InuA inhibited cancer cell growth and induced cell cycle arrest and apoptosis by inhibiting the NFAT1-MDM2 pathway and modulating the expression of proteins involved in cell cycle progression, apoptosis, and DNA damage response. ATR, Ataxia telangiectasia and Rad3 related; Bax, Bcl2-Associated X Protein; Bcl2, B-cell lymphoma 2; Cdk2, Cyclin-dependent kinase 2; Cdk4, Cyclin-dependent kinase 4; Cdk6, Cyclin-dependent kinase 6; Chk1, Checkpoint kinase 1; Chk2, Checkpoint kinase 2; InuA, Inulanolide A; MDM2, mouse double minute 2; NFAT1, nuclear factor of activated T cells 1; PARP, poly ADP ribose polymerase; p-Chk1, phosphorylated Chk1; p-Chk2, phosphorylated Chk2; p-p53, phosphorylated p53; p-H2AX, phosphorylated H2A histone family, member X.

In the present study, we evaluated the anticancer efficacy of a new dual NFAT1-MDM2 inhibitor InuA and examined the possible molecular mechanisms for its activities in preclinical models of human breast cancer *in vitro* and *in vivo*. We have demonstrated several important points in the present study. First, InuA exhibited a potent cell growth-inhibition activity *in vitro* in a wide spectrum of human cancer cell lines, with minimal cytotoxicity against normal cells. Second, InuA inhibited breast cancer cell proliferation and induced cell cycle arrest at G2/M phase and apoptosis *in vitro*, in concentration-dependent and p53-independent manners. Third, InuA suppressed the growth of orthotopic tumors in mice, without inducing any host toxicity. Fourth, InuA inhibited cell migration and invasion *in vitro* and prevented tumor metastasis to lungs *in vivo*. Finally, InuA inhibited the MDM2, NFAT1 and proteins associated with cell proliferation, and activated apoptotic signal related proteins both *in vitro* and *in vivo*, contributing to the anticancer activity of the compound. Collectively, Figure 8 depicted the possible mechanisms of action of InuA against breast cancer cells, based on the aforementioned *in vitro* and *in vivo* findings.

We first investigated the sensitivity of various normal and cancer cell lines to InuA treatment and this compound exhibited varying cytotoxicity against a broad spectrum of cancer cell lines, regardless of p53 status. Interestingly, the cancer cells containing wild-type p53 (MCF7 and HCT116) showed slightly more sensitive than their corresponding p53 KD or knockout (KO) cell lines (Figure 1), suggesting that the anticancer effects of InuA might not be completely independent of p53. In p53 wild-type cells, InuA treatment decreased MDM2 expression, resulting in p53 activation and inducing both p53-dependent and -independent cell death; In p53-KD, -mutant, or -null cells, InuA treatment only caused p53-independent anticancer effects, leading to higher IC_50_ values. Of note, all the used breast cell lines were highly sensitive to the treatment of this compound. Two breast cancer cell lines MCF7 (p53 wild-type, ER positive) and MDA-MB-231 (p53 mutant, triple negative) were selected based on their genetic backgrounds for investigating the *in vitro* activity of InuA and its molecular mechanisms. Both breast cancer cell lines were responsive to the treatment of InuA, showing inhibition in cell proliferation and activation in cell cycle arrest and apoptosis, regardless the status of p53 and ER. Given the excellent anticancer activity of the compound, we further demonstrated its inhibitory effects on NFAT1 and MDM2 in both cell lines. In addition, the changes in various proteins associated with cell proliferation, cell cycle progression, apoptosis, and DNA damage response signaling pathways further corroborated the effects of NFAT1 and MDM2 inhibition. The important role of NFAT1-MDM2 pathway in InuA's anticancer activity has also been validated in both NFAT1 KD and MDM2 KD breast cancer cell lines (both p53 wild-type and mutant). Both NFAT1 KD and MDM2 KD led to decreased sensitivity to InuA, indicating loss of molecular targets (NFAT1 and MDM2) for InuA reduced the target cells’ sensitivity. However, further exploration for precise molecular mechanisms responsible for the anticancer activity of InuA also needs to be considered.

To provide more clinically relevant animal models for *in vivo* testing, we developed MDA-MB-231 orthotopic mouse model, which is better at reflecting the original situation and predicting the efficacy of the drugs than subcutaneous xenograft tumor models. In our studies, InuA showed substantial inhibitory effects on tumor growth. Furthermore, a significant increase in apoptotic cells was observed in InuA-treated tumors, as demonstrated by TUNEL staining, which was consistent with that was observed in *in vitro* studies. Not surprisingly, the expression patterns of NFAT1, MDM2, and related proteins in InuA-treated tumors were similar as seen in *in vitro* assays, providing a basis for future biomarker studies for clinical translation of this compound.

With regard to the preventive effects of InuA on breast cancer metastasis, our *in vitro* studies indicated that the compound at sublethal concentrations effectively inhibited breast cancer cell migration and invasion, as illustrated by wound-healing assay and transwell invasion assay, respectively. The *in vivo* results obtained from orthotopic mouse model further supported the potent preventive effects of InuA on tumor metastasis. Although InuA exhibited inhibitory effects on cancer cell motility, it could be partially responsible for the reduced lung metastasis *in vivo*. The effects of InuA on angiogenesis also need to determine in the future study. More sensitive assays and more clinically relevant metastasis models are expected for further preclinical evaluation and clinical translation of this compound.

Of note, our *in vitro* and *in vivo* results showed a favorable safety profile of InuA in the preclinical breast cancer models in the present study. In the cell cytotoxicity assays, we found that the normal cell lines were less sensitive to the treatment of InuA in comparison to the cancer cell lines, indicating that InuA had selective cytotoxicity against cancer cells. In the *in vivo* efficacy studies, we demonstrated that the average body weights of InuA-treated mice were comparable with that of vehicle-treated mice and no organ abnormalities were observed in the histological examinations, implying that InuA did not induce any host toxicity at the effective dose for suppression of breast tumor growth. However, considering that NFAT1 plays an important role in human immune system, which is a relevant point for using NFAT1 inhibitor in the clinics, the potential adverse effects of InuA should be examined in immune cells. In order to get results that are more relevant to the clinics, development of a syngeneic orthotopic model in mice that have immune system is necessary. A thorough understanding of the pharmacological effects and mechanisms of action of InuA is certainly needed for the development of this novel anticancer agent. Further pharmacological and toxicological studies are needed to determine the oral bioavailability, toxicity profile, optimal therapeutic dose and route of the compound. More clinically relevant models of human cancer should be utilized to determine the efficacy, safety, and molecular mechanisms of InuA.

Based on our preliminary data, dual NFAT1-MDM2 inhibitors (JapA and InuA) have desirable efficacy for further preclinical studies and development as clinical candidates. Both compounds are dimeric sesquiterpene lactones and have an identical monomeric fragment. Our initial SAR analyses have indicated that this identical monomeric fragment, especially the α-methylene-γ-lactone group in this fragment, is crucial for their anticancer activity. However, more detailed SAR analyses are still needed to delineate the role of functional groups in the anticancer activity in the future studies.

In summary, the present study has demonstrated that InuA is a novel dual inhibitor of NFAT1-MDM2 pathway and has potent inhibitory effects on breast cancer cell growth and metastasis *in vitro* and *in vivo*. These results together suggest that InuA may serve as a potential therapeutic agent for breast cancer treatment and prevention and benefit the patients with advanced disease.

## MATERIALS AND METHODS

### Chemicals and reagents

All chemicals and solvents used in the present study were of highest grade available. InuA with a purity higher than 95% was prepared as reported previously [34]. The antibodies against human MDM2 (Ab-2), p21 (Ab-1), and p-H2AX (Ser139) were bought from EMD Chemicals (Gibbstown, NJ, USA). The antibodies against human p53 (DO-1), Cdk2 (M2), Cdk4 (H-22), Cdk6 (C-21), cyclin D1 (DCS-6), cyclin E (HE12), c-Myc (0.N.222), Bax (N-20), Bcl-2 (100), PARP (H-250), Chk1 (G-4), Chk2 (B-4), and ATR (N-19) were purchased from Santa Cruz Biotechnology (Santa Cruz, CA, USA). The antibodies against human p-Chk1 (Ser317), p-Chk2 (Thr68), and p-p53 (Ser15) were obtained from Cell Signaling Technology (Danvers, MA, USA). The anti-human NFAT1 (1/NFAT-1) antibody was sourced from BD Biosciences (San Jose, CA, USA) and the anti-human β-actin (AC-15) antibody was purchased from Sigma (St. Louis, MO, USA). The goat anti-mouse IgG (H+L) and goat anti-rabbit IgG (H+L) antibodies were bought from Bio-Rad (Hercules, CA, USA). The NFAT1 and MDM2 siRNA pool and control siRNA pool were obtained from Thermo Scientific (Rockford, IL, USA).

### Cell lines and culture conditions

Human normal and cancer cell lines were obtained from American Type Culture Collection (Rockville, MD, USA). All cell culture media mentioned below were supplemented with 10% fetal bovine serum (FBS) (Atlanta Biologicals, Lawrenceville, GA, USA) and 1% penicillin/streptomycin (Corning, Manassas, VA, USA), except for those indicated otherwise. Human breast cancer MCF7, MCF7 p53 KD, MDA-MB-468, and MDA-MB-231 cells and human breast epithelial MCF10A cells were cultured as described previously [21, 26]. Human prostate cancer PC3 cells were maintained in Ham's F12 medium containing 2 mM L-glutamine, LNCaP cells were cultured in RPMI 1640 media containing 2 mM L-glutamine, 10 mM HEPES, 1 mM sodium pyruvate, glucose (4.5 mg/mL), and sodium bicarbonate (1.5 mg/mL), and DU145 cells were cultured in RPMI 1640 medium. Human lung cancer A549 cells were grown in Ham's F12 medium and H385 cells were cultured in RPMI 1640 medium. Human pancreatic cancer HPAC cells were grown in DMEM/Ham's F12 media containing 1.2 g/L sodium bicarbonate, 2.5 mM L-glutamine, 15 mM HEPES, and 0.5 mM sodium pyruvate supplemented with 2 μg/mL insulin, 5 μg/mL transferrin, 40 ng/mL hydrocortisone, 10 ng/mL epidermal growth factor, and 5% FBS, Panc-1 cells were cultured with RPMI 1640 containing 1 mM HEPES buffer, 25 μg/mL gentamicin, 1.5 g/L sodium bicarbonate, and 0.25 μg/mL amphotericin B, and BxPC3 were grown in RPMI 1640 medium. Human colon cancer HCT116 and HCT116 p53KO (HCT116 p53^−/−^) cells were grown in McCoy's 5A medium. Human osteosarcoma U2OS cells were maintained in DMEM medium. Human ovarian cancer SKOV-3 cells were maintained in McCoy's 5A medium and OVCAR-3 cells were cultured in RPMI 1640 medium supplemented with 20% FBS, 10 mM of sodium pyruvate, 10 mM of HEPES, 10 mg/L of bovine insulin, and 4.5 g/L of glucose. Human liver cancer HepG2 and Hep3B cells were grown in DMEM medium. Human glioma U87MG cells were grown in Eagle's minimum essential medium (EMEM). Human embryonic kidney HEK293 cells were grown in DMEM medium. The inducible MDM2 KD MCF7 cell line was established previously [21, 26] and was grown in DMEM medium containing 0.5 μg/mL puromycin (Sigma, St. Louis, MO, USA).

### Assays for *in vitro* anticancer activity of InuA

The assays for the effects of InuA on cell viability (MTT assay) [21, 26], cell proliferation [bromodeoxyuridine (BrdU) incorporation assay] [21, 26], cell cycle progression [21, 26], cell apoptosis (Annexin V-FITC apoptosis detection kit) [21, 26], cell migration (wound healing assay) [21, 35], and cell invasion (transwell invasion assay) [36] were performed as described in our previous studies.

In brief, the cells were seeded in 96-well plates (3–4 × 10^3^ cells/well) for 24 h and exposed to various concentrations of InuA (0–50 μM) for another 72 h for the MTT assay. For BrdU incorporation assay, the cells (5–8 × 10^3^ cells/well) in 96-well plates were treated with various concentrations of InuA (0, 2.5, 5, and 10 μM) for 14 h, and then incubated with the BrdU for another 10 h. To detect cell apoptosis, the cells in 6-well plates (2–3 × 10^5^ cells/well) were exposed to various concentrations of InuA (0, 2.5, 5, and 10 μM) for 48 h. The cells were then harvested and resuspended in Annexin V binding buffer and incubated with Annexin V-FITC and propidium iodide (Sigma, St. Louis, MO, USA). Both early apoptosis and later apoptosis were determined by flow cytometry (BD Biosciences, San Jose, CA, USA). To analyze the cell cycle distribution, the cells in 6-well plates (2–3 × 10^5^ cells/well) were treated with various concentrations of InuA (0, 2.5, and 5 μM) for 24 h. Then the cells were fixed at 4^°^C overnight, incubated with RNase (Sigma, St. Louis, MO, USA), and stained with propidium iodide for flow cytometry analysis.

To determine the effects of InuA on cell migration, the monolayer of MDA-MB-231 cells was grown to confluence in 6-well plates and scratched in each well using a pipette tip. The cells were then treated with InuA (1 or 2 μM), and five fields of each wound were monitored and photographed at 0, 8, and 24 h. To determine the effects of InuA on cell invasion, the cells were seeded on the upper well of Boyden chamber (1–2 × 10^4^ cells/well) and exposed to InuA (1 or 2 μM) for 24 h. The cells on the upper surface were then removed by wiping with a cotton swab and the cells adhering to the filter were stained with Mayer's Hematoxylin and Eosin solution and photographed under a phase-contrast Olympus microscope (Olympus America Inc, PA, USA). The positive staining area was measured using image analysis software.

### Western blotting analysis

In brief, the cells in 6-cm dishes (3–4 × 10^5^ cells/dish) were exposed to various concentrations of InuA (0, 2.5, 5, and 10 μM) for 24 h. Then the cells were lysed in NP-40 lysis buffer containing a protease inhibitor mixture (Sigma, St. Louis, MO, USA). The tumor tissue samples were homogenized in NP-40 lysis buffer and centrifuged for the supernatants. Both cell and tumor tissue samples were quantified and subjected to Western blotting analysis for the expression levels of various proteins using the methods as described previously [21, 26].

### NFAT1 and MDM2 silencing

MCF-7 and MDA-MB-231 cells (2 × 10^5^ cells/dish) were transfected with NFAT1 or MDM2 siRNA pool or control siRNA pool as described previously [21, 33]. After 36 h of transfection, cells were treated with InuA (0, 2, 5, and 10 μM) for 24 h. The cell lysates were used for Western blotting analysis. For cell viability assay, the transfected cells were seeded in 96-well plates (3 × 10^3^ cells/well) and treated with InuA (0–10 μM) for 72 h for MTT assay.

### MDA-MB-231 orthotopic model and *in vivo* efficacy study of InuA

The animal protocols were approved by the Institutional Animal Use and Care Committee of Texas Tech University. Female athymic nude mice (nu/nu, 4-6 weeks) were purchased from Charles River Laboratories International, Inc. (Wilmington, MA, USA). After anesthesia, MDA-MB-231 cells (1 × 10^6^ cell/mouse) were implanted into the second thoracic mammary fat pad. After two weeks, tumor-bearing mice were divided into control and treatment groups (10 mice/group). InuA was dissolved in PEG400:ethanol:saline (57.1:14.3:28.6, v/v/v) and administered by intraperitoneal injection at a dose of 30 mg/kg/d, 5 d/wk for 24 days. The control group received the vehicle only. All mice were monitored for activity, physical condition, body weights, and tumor growth during the treatment period. The tumor volume (mm^3^) was calculated by the formula: (a × b^2^)/2, where “a” is the long diameter (cm) and “b” is the short diameter (cm). At the end of the experiments, all mice were examined for tumor metastasis to lungs. Various tissues (lungs, brain, heart, liver, spleen, and kidneys) were removed and processed for Hematoxylin and Eosin (H&E) staining, and orthotopic tumors were removed and processed for Western blotting analysis, immunohistochemistry, and TUNEL assay.

### Immunohistochemistry, TUNEL staining, and H&E staining

In brief, tumors and various tissues from the mice bearing orthotopic tumors were fixed in 10% formalin and embedded in paraffin. Then the tumor and tissue sections (5 μm) were prepared and deparaffinized in xylenes, rehydrated, and washed with PBS. The immunohistochemistry examination of NFAT1 and MDM2 was performed using the biotinylated anti-human NFAT1 and MDM2 antibodies (diluted 1:50 in 5% BSA in PBS), respectively [21, 26, 36]. Sections were counterstained with hematoxylin and mounted. The TUNEL assays were performed as described previously [21, 26, 36]. Briefly, tumor sections were incubated with proteinase K for 15 min, and then DNA breaks were labeled with terminal deoxytransferase (TdT) in a humidified chamber for 5 min. The sections were then incubated with 50 μL of biotinylated BrdU antibody for 1 h. Both the immunohistochemistry and TUNEL staining were developed with streptavidin-conjugated HRP and 3,3′-diaminobenzidene (HRP substrate). For H&E staining, tissue sections were stained in Mayer's Hematoxylin for 10 min and then stained with Eosin for 1 min. All the slides were analyzed and viewed under a phase-contrast Olympus microscope (Olympus America Inc., Central Valley, PA).

### Statistical analysis

Quantitative data were presented as mean ± SEM from at least three independent experiments. Statistical significances were determined using Student's *t*-test and *P* < 0.05 was considered to be statistically significant.
